# Proteoform Profiles Reveal That Alpha-1-Antitrypsin in Human Serum and Milk Is Derived From a Common Source

**DOI:** 10.3389/fmolb.2022.858856

**Published:** 2022-02-22

**Authors:** Shelley Jager, Dario A. T. Cramer, Max Hoek, Nadia J. Mokiem, Britt J. van Keulen, Johannes B. van Goudoever, Kelly A. Dingess, Albert J. R. Heck

**Affiliations:** ^1^ Biomolecular Mass Spectrometry and Proteomics, Bijvoet Center for Biomolecular Research and Utrecht Institute for Pharmaceutical Sciences, University of Utrecht, Utrecht, Netherlands; ^2^ Netherlands Proteomics Center, Utrecht, Netherlands; ^3^ Department of Pediatrics, Vrije Universiteit, University of Amsterdam Emma Children’s Hospital, Amsterdam UMC, Amsterdam, Netherlands

**Keywords:** alpha-1-antitrypsin, proteoforms, allotypes, proteogenomics, mammary gland, native mass spectrometry, post-translational modifications

## Abstract

The Alpha-1-Antitrypsin (A1AT) protein is an important protease inhibitor highly abundant in human serum and other body fluids. Additional to functioning as a protease inhibitor, A1AT is an important acute phase protein. Here, we set out to compare the proteoform profiles of A1AT purified from the human serum and milk of eight healthy donors to determine the origin of human milk A1AT. Following affinity purification, size-exclusion chromatography coupled to native mass spectrometry was used to monitor individual proteoform profiles comparing inter- and intra-donor profiles. The A1AT intra-donor proteoform profiles were found to be highly identical between serum and milk, while they were highly distinct between donors, even when comparing only serum or milk samples. The observed inter-donor proteoform variability was due to differences in the abundances of different *N-*glycoforms, mainly due to branching, fucosylation, and the relative abundance of N-terminally processed A1AT fragments. From our data we conclude that nearly all A1AT in serum and milk is synthesized by a common source, i.e. the liver, and then secreted into the circulation and enters the mammary gland *via* diffusion or transport. Thereby, proteoform profile changes, as seen upon infection and/or inflammation in the blood will be reflected in the milk, which may then be transferred to the breastfed infant.

## Introduction

The protein Alpha-1-Antitrypsin (A1AT or SerpinA1) is best known as a serine protease inhibitor and inhibitor of neutrophil elastase in the lungs (Greene et al., 2016). However, it is also an acute phase protein with immunomodulatory and anti-inflammatory abilities (Poller et al., 1999; Crowther et al., 2004). Upon infections A1AT serum concentrations increase and its proteoform profile can also adapt drastically (Smith et al., 2013; Ruhaak et al., 2014; Mondal et al., 2016; Kobayashi et al., 2019; Lechowicz et al., 2020; Yin et al., 2020), due to changes in post translational modifications (PTM), mainly *N-*glycosylation and N-terminal processing. A1AT has been shown to inhibit to some extent viral infections by HIV type 1, Influenza, and more recently also by SARS-CoV-2 (Guttman et al., 2015; Lechowicz et al., 2020; Azouz et al., 2021). Additionally, A1AT can act to reduce inflammation *via* inhibition of IL-8 and both TNF-alpha receptors (with weak affinity), while also increasing the expression of the anti-inflammatory cytokines IL-1Ra and IL-10 (Tilg et al., 1993; Bergin et al., 2010; Bergin et al., 2014). While there is extensive evidence of the biological function of A1AT in serum, the main reported functionality in human milk is as a protease inhibitor, both in the mammary gland and in infant digestion (Chowanadisai and Lönnerdal, 2002). However, with the known serum anti-inflammatory and immune-modulatory mechanisms of A1AT, it can be assumed that the importance of A1AT in human milk may also extend beyond anti-proteolytic functions.

Hepatocytes in the liver have been reported to be the main site of A1AT production (Greene et al., 2016), however, it has also been reported that A1AT may also be synthesized in the mammary gland after which this pool of A1AT is excreted into human milk (Elwakiel et al., 2019). It has so far not been established whether all A1AT in human milk is synthesized locally in the mammary gland or if part or all of A1AT is originating from the serum and gets into the milk *via* diffusion and/or transport (Chowanadisai and Lönnerdal, 2002).

Alpha-1-antichymotrypsin (AACT or SerpinA3) and A1AT are the most abundant protease inhibitors found in human milk, with concentrations ranging from 0.4 to 0.7 mg/ml and 0.1–0.4 mg/ml, respectively (Chowanadisai and Lönnerdal, 2002). Both proteins are within the top 25 most abundant proteins in milk (Table 1) (Elwakiel et al., 2019). The abundance of both protease inhibitors decreases throughout lactation (Zhu et al., 2021). Notably, Chowanadisai and Lönnerdal reported that AACT purified from human milk and serum differ substantially in their proteoforms, revealed by differences in mobility by gel electrophoresis and the relative distribution of antichymotrypsin bands analyzed by western blot analysis (Chowanadisai and Lönnerdal, 2002), possibly reflecting the different sources of their origin. As far as we know, such data on A1AT is not yet available.

**TABLE 1:** Reported average concentrations of A1AT and AACT in human serum and milk.

	Human serum	Human milk
Average A1AT concentration	1.3 mg/ml	0.25 mg/ml
Average AACT concentration	0.4 mg/ml	0.55 mg/ml
Total protein concentration	60–80 mg/ml	85–126 mg/ml
%A1AT of total protein	1.6–2.2%	0.2–0.3%
%AACT of total protein	0.5–0.7%	0.4–0.6%

The quoted numbers come from references (Busher, 1990; Chowanadisai and Lönnerdal, 2002; Hollander et al., 2007; Verd et al., 2018) ***.***

From the mother’s or the infant’s perspective, the immunomodulatory and anti-inflammatory properties of A1AT and its role as an acute phase protein in human milk are understudied. A1AT is known to play an important role in the digestion of milk proteins in the infant’s gastrointestinal tract (Lönnerdal, 2003). For example, *in vitro* experiments have shown that the addition of A1AT to human milk results in lower degradation rates of lactoferrin by pancreatin, thus A1AT protects lactoferrin from digestion (Chowanadisai and Lönnerdal, 2002). This is important as lactoferrin has anti-microbial and immune-regulatory properties. The protection of other proteins against degradation is likely an important function of A1AT in human milk, due to the need for many human milk proteins to survive infant digestion to exhibit functionality in the more distal part of the gastrointestinal tract. This sort of data suggests that A1AT might have different functions in milk than in serum.

**FIGURE 1:**
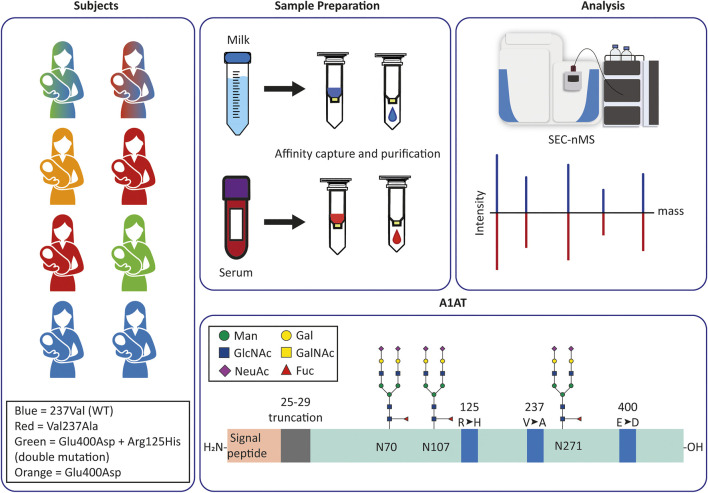
Experimental design and set-up to monitor and compare personalized proteoform profiles of A1AT from serum and milk. **(A)** Milk and serum were collected from eight donors. The colors of each donor correspond to the donor’s A1AT genotype: blue is M1V (237Val), red is M1A (Val237Ala), green is M2 (Arg125His + Glu400Asp), orange is M3 (Glu400Asp); double-colored figures represent donors heterozygous for A1AT. Thus, green and blue is heterozygous M1VM2, and red and blue is heterozygous M1AM1V. **(B**,**C)** A1AT was captured from serum or milk using immuno-affinity beads, the collected A1AT was subsequently further purified by using a SEC column coupled to a high-resolution mass spectrometer. The acquired data was deconvoluted to zero-charge to provide an individual mass profile where peaks represent different proteoforms. These profiles can then be compared within a single donor between serum and milk, or between donors in serum or milk. **(D)** Schematic overview of the A1AT protein, with in orange the (cleaved) signal peptide and in green the mature circulating protein. Indicated are the three N-glycosylation sites, and the mutation hotspots (blue) defining the different genotypes. Indicated in grey is the 5 amino acid stretch that is occasionally missing in a N-terminal truncated form of A1AT. The glycan structures represent the most common glycoforms, an overview of other prevalent glycoforms can be found in Supplementary Figure S1.

Since we hypothesized that A1AT from serum and milk may have different origins and functions, we set out to address the question whether there are also differences in their proteoform profiles. Differences in the origin of A1AT, either in the liver or the mammary gland, are expected to lead to differential glycosylation patterns as certain glyco-enzymes may be more/less active in hepatocytes than in the mammary gland. Moreover, further local action on the A1AT proteins, for instance *via* N-terminal processing by local proteases and/or peptidases may also lead to differences in proteoform signatures between serum and milk A1AT. To address this question, we purified A1AT using immunoaffinity chromatography, and profiled the proteoform profiles of serum and milk A1AT from eight donors, whereby milk and serum were donated at (nearly) the same time, using on-line size exclusion chromatography high-resolution native mass spectrometry (SEC-nMS) (*see* Figure 1).

## Materials and Methods

### Donor Population

Human serum and milk samples were collected from eight healthy lactating women who delivered at the Amsterdam UMC and who participated in our previous study (van Keulen et al., 2021). Ethical approval was obtained from the Medical Ethics Committee of the Amsterdam UMC, location VUmc and written informed consent was obtained from all participants. Donors were instructed to collect 100 ml of human milk in a sterile provided bottle and to store it in their freezer until collection by the study staff during a home visit. During this visit, maternal serum was collected by a trained phlebotomist. Serum and milk samples were stored at −80°C prior to analysis.

### Purification of Alpha-1-Antitrypsin From Serum

Unless stated otherwise, all other chemicals and proteins were purchased from Sigma-Aldrich (Saint Louis, MO, United States). Serum samples were filtered using a 0.22 µm filter (WAT200516 Acrodisc; Waters, Milford, MA, United States), aliquoted, and then stored at −80°C until use. Empty spin columns (Pierce^™^ Spin Cups - Cellulose Acetate Filter; Thermo-Fisher Scientific, Waltham, MA, United States) were conditioned with 2 × 100 µl PBS (800 × *g*, 60 s, RT) followed by addition of 20 µl antitrypsin affinity resin (CaptureSelect Alpha-1 Antitrypsin Affinity Matrix; Thermo-Fisher, Waltham, MA, United States) and 25 µl PBS and then centrifuged (80 × *g*, 30 s, RT). The affinity matrix consists of a non-disclosed antibody fragment having high affinity for A1AT, coupled to the agarose beads *via* a flexible linker. Subsequently, 100 µl serum was added per column, incubated for 1 h at RT, and centrifuged (80 × *g*, 30 s, RT; and then 800 × *g*, 60 s, RT). Next, the column underwent serial washing with PBS (3 × 50 μl, 2 × 100 µl). Purified A1AT was eluted from the affinity matrix with Glycine (0.1 M, pH 3.0) in three subsequent steps (2 × 50 μl, 1 × 25 µl) into collection vials containing 20 µl TRIS/HCl (1 M, pH 8.5). The columns were centrifuged (80 × *g*, 30 s, RT; and then 800 × *g*, 60 s, RT) after each washing and collection step. Finally, the collected A1AT was stored at −20°C until further use.

### Purification of Alpha-1-Antitrypsin From Skimmed Human Milk

Human milk samples were thawed on ice, to minimize proteolytic degradation of the milk proteins. Once thawed, samples were defatted by centrifuged (1500 × *g*, 20 min, 4°C), after which the water fraction was transferred to a new vial. The process was repeated, and the resulting skimmed milk was aliquoted and stored at −80°C until further use. A1AT was captured, purified and eluted from the skimmed milk samples in the same manner as serum samples described above. For skimmed milk samples the starting volume was around 200 µl.

### Size-Exclusion Chromatography Coupled to Native MS Analysis of Purified A1AT

Purified A1AT from human serum or milk samples, 20–30 and 200 µl respectively, was buffer exchanged into 100 mM aqueous ammonium acetate (AMAC) (pH 7.2) by ultrafiltration with a 10 kDa cut-off filter (vivaspin500; Sartorius Stedim Biotech, Göttingen, Germany). The final volume was 15–30 µl. The SEC-nMS analysis was performed by using an Agilent 1290 infinity HPLC system (Agilent Technologies, Amstelveen, Netherlands) coupled online to a modified Exactive Plus Orbitrap instrument with extended mass range (EMR) (Thermo Fisher Scientific, Bremen, Germany). An ACQUITY UPLC Protein BEH SEC 200 Å Column (4.6 × 300 mm, 1.7 µm particle size; Waters, Milford, MA, United States) was used, 100 mM solution of Ammonium Acetate (pH 7.2) in MilliQ was used as the eluent, the autosampler temperature was 7°C and the injection volume was optimized per sample. The flowrate was set to 0.2 ml/min. An in-house made split flow was used to couple the SEC column to the electrospray ionization (ESI)-MS, ensuring a flow rate of 7 μl/min directed to the MS. All chromatograms and mass spectra were obtained with the same MS parameters in positive ion mode with the spray voltage at 2 kV, the in-source collision induced dissociation to 20 eV and the collision energy to 30 eV. The source temperature was 250°C. The mass spectra were acquired in a *m/z* range of 500–15,000. Transient acquisition time was set to a resolution of 17,500 at an *m/z* of 200.

### SEC-nMS Data Analysis

Native mass spectra were deconvoluted to a zero-charge spectrum using Intact Mass software from Protein Metrics (version 4.0-43 × 64). The timeframe selected was between 12.5 and 13.5 min. Used deconvolution settings were: mass range 50,000–55,000 Da; m/z range 200–4500 min difference between peaks 7 Da, iteration 20–30, charge range 5–20.

To compare proteoform profiles Pearson correlations were calculated between zero-charge spectra in the mass range 50–53 kDa using 7 Da bins. Correlation values were clustered using hierarchical clustering using Euclidean distance and ward linkage. The dendrogram was visualized using iTOL (Letunic and Bork, 2021).

## Results

Here we set out to address the question whether human serum and milk A1AT originate from the same or different origins. Therefore, we developed a method to capture A1AT and profile the personalized proteoform profiles using an SEC-nMS approach (Figure 1). Milk and serum were collected from healthy donors, roughly at the same time, and A1AT was purified using immuno-affinity beads (i.e. CaptureSelect Alpha-1 Antitrypsin Affinity Matrix). The purified A1AT was subjected to online coupled SEC-nMS. The subsequent recorded high-resolution native mass spectra were deconvoluted to zero charge yielding well-resolved mass plots, from which individuals A1AT proteoforms could be identified and quantified. We observed that online coupled SEC-nMS provided an edge over direct infusion native MS as not only the sample introduction system was more robust, but it also provided the separation of co-purified contaminant protein lactoferrin (in the human milk samples) from A1AT through SEC, preventing overlapping ion signals in the mass spectra.

### Method Optimization

Firstly, the method for A1AT capturing using affinity resins was optimized for human serum. The affinity resin consisted of agarose beads covalently linked to an antibody-fragment with high affinity for A1AT. In short, serum was incubated with the affinity matrix, subsequent washing steps were performed to remove low binding proteins, followed by an elution step to collect the purified A1AT protein. Optimization of the elution buffer led to the use of a glycine solution with a pH of 3, which was directly neutralized in the collection vial using TRIS/HCl with a pH of 8.5 to prevent the observed denaturation of A1AT during storage. The serum-to-resin-ratio was also optimized, as excessive amounts of resin led to non-specific binding by other proteins, for example albumin. However, in analyzing the human milk samples we were unable to achieve a single optimal milk-to-resin-ratio as the A1AT concentration in human milk decreases by 2–3-fold throughout lactation. Consequently, impurities resulting from non-specific binding proteins could not be prevented entirely when analyzing the milk samples.

**FIGURE 2:**
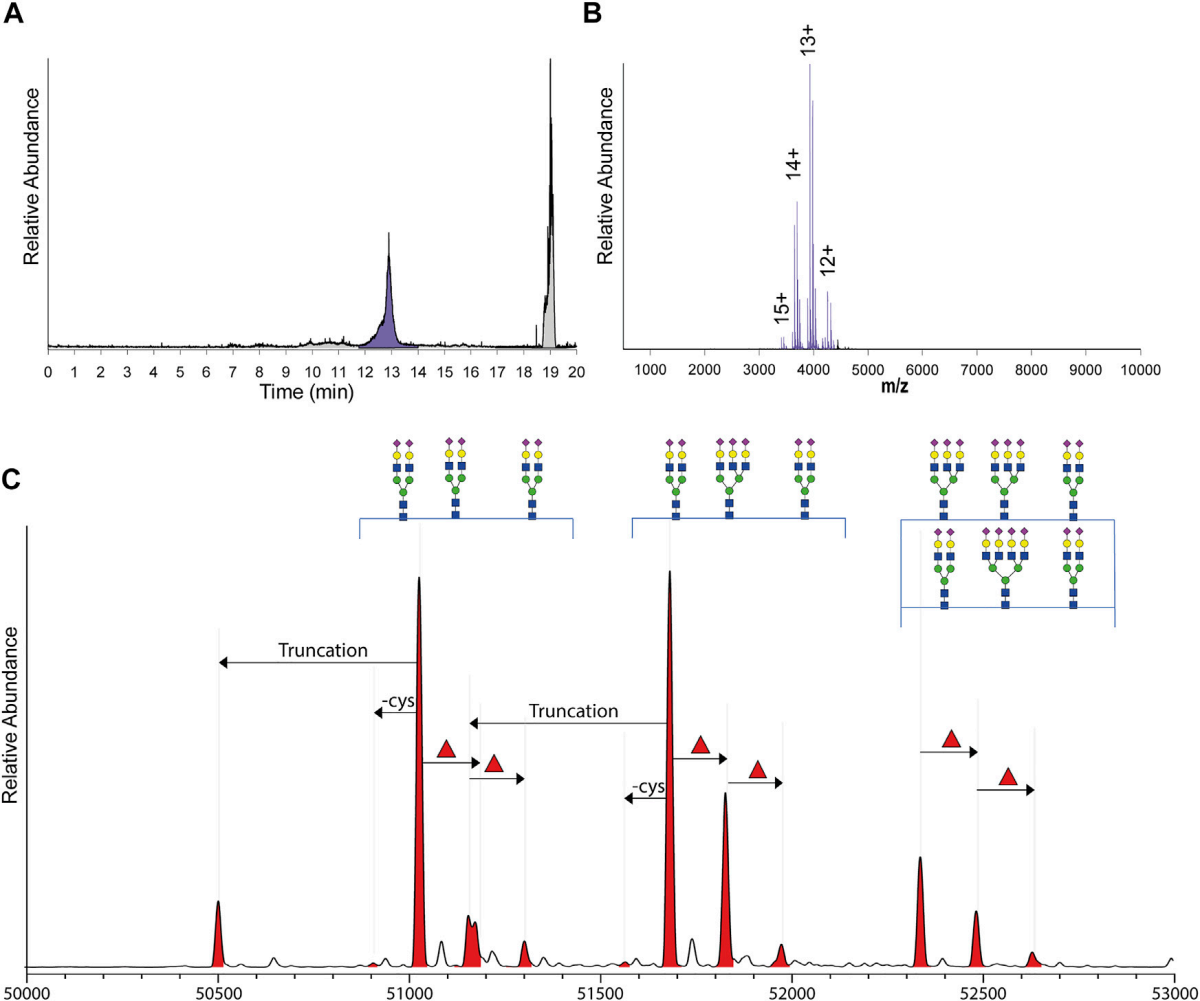
From SEC fractionation of immune-purified A1AT to annotated qualitative and quantitative proteoform profiles. **(A)** Typical total ion current (TIC) chromatogram obtained by SEC-nMS following injection of immune-purified serum or milk A1AT, with the fraction containing A1AT highlighted in purple and all other fractions in grey. **(B)** Typical, non-processed native ESI-spectrum of A1AT with observed charge states annotated. **(C)** The zero-charge mass deconvoluted spectrum revealing mass peaks between 50 and 53 kDa all annotated as different A1AT proteoforms. The main peaks are annotated by their glycan composition and indicated with arrows are the additional annotated PTMs: the N-terminal truncation (truncation), decysteinylation of C256 (-cys), and additional fucoses (red triangle) which can either be located on the antennae or on the glycan core.

In human milk, following the A1AT targeted immune-purification, the major non-specific binding protein was found to be lactoferrin, which is a 80 kDa protein. Due to the subsequent use of the SEC column, A1AT and lactoferrin could be separated based on their size (Figure 2A). A standard of commercial lactoferrin (0.4 mg/ml) and commercial A1AT (0.4 mg/ml) confirmed that lactoferrin and A1AT eluted on the used column and conditions separately between 10.8–11.8 and 12.5–13.5 min, respectively.

The raw SEC-nMS spectra revealed mostly ions originating from different proteoforms of A1AT, spanning four charge states, ranging from [M+12H]^12+^ to [M+15H]^15+^ (Figure 2B). The high-resolution native mass spectra were deconvoluted to zero charge to provide a mass plot with well-separated signals for all different proteoforms with corresponding abundances. Proteoforms were assigned based on the known A1AT amino acid sequence (and thus peptide backbone mass) combined with earlier reported data on typical post-translational modifications occurring on A1AT (Kolarich et al., 2006; Clerc et al., 2016; Yin et al., 2018; Lechowicz et al., 2020). In more detail, A1AT harbors three *N-*glycosylation sites and a free cysteine that can also be cysteinylated, the *N-*glycans can be branched and fucosylated, and also a minor portion of A1AT can be further processed and harbors a truncated N-terminus (Supplementary Figure S1). Using the theoretical mass shifts of all these modifications, all abundant peaks in the measured proteoform profiles could be assigned and quantified (Supplementary Table S1).

#### Comparing Proteoform Profiles of A1AT Between Milk and Serum

The analyzed sample set consisted of samples obtained from eight healthy donors. The mean age of the mothers ranged from 30 to 38 years (mean 34.4 ± 2.6) with a range in gestation between 39 and 41 weeks (mean 40.4 ± 0.7) (Supplementary Table S2). For five of the individual donors, serum and milk were collected on the same day, in two of the donors there was 1 day between and for one donor there were 2 days between the collections, whereby the collection of the milk sample always occurred prior to the serum sample.

In all samples, the fraction between 12.5 and 13.5 min contained exclusively A1AT (Figure 2A); except for the milk samples from donors 2 and 7. In the milk sample of donor 2, an unknown contaminant of 51,201.00 Da could be identified and in the milk sample of donor 7, several unidentified proteins co-eluted. This was probably the result of a relatively low A1AT concentration in this particular donor, resulting from the fact that the milk and serum samples from donor 7 were taken quite late in the lactation phase, i.e. at 40 weeks postpartum. It has been described that the A1AT concentration in human milk declines throughout lactation (Elwakiel et al., 2019; Zhu et al., 2021). This low A1AT concentration probably resulted in the non-specific binding by other contaminant proteins to the affinity resin.

**FIGURE 3:**
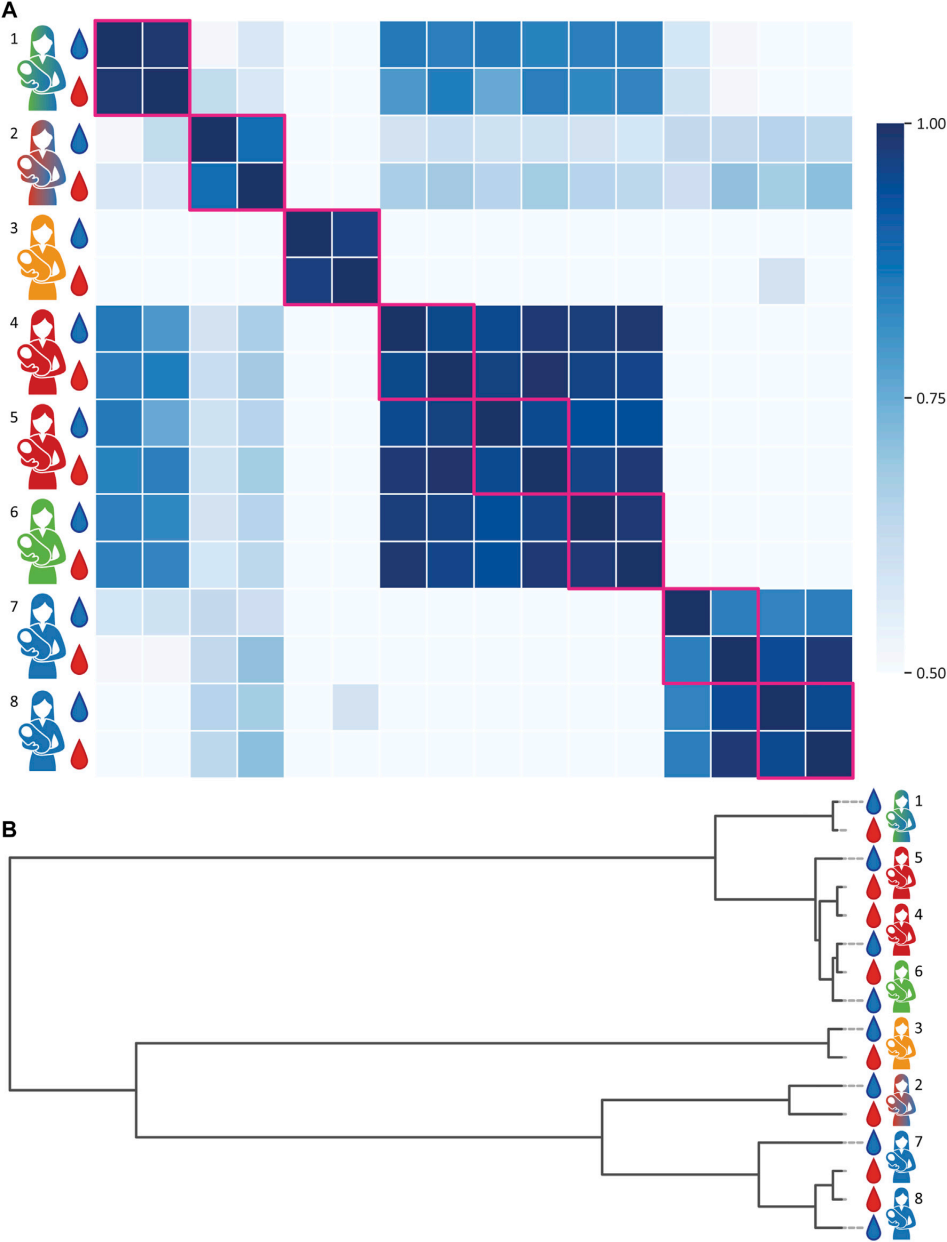
Proteoform profile correlations of A1AT obtained from paired milk and serum samples of eight individual donors. Donors and samples are color-coded as in Figure 1, milk and serum are depicted as blue and red droplets, respectively. **(A)** A correlation matrix of all samples. Within the same donor the A1AT proteoform profiles of serum and milk show a very high correlation. Donors of the same genotype, or heterozygous genotypes with common alleles, tend to have a higher inter-donor correlation. Milk and serum of donors 2 and 7 have a slightly lower intra-donor relation, due the presence of an unknown protein contaminant in the milk sample. When disregarding the 51,201.00 Da contaminant in the milk sample of donor 2, the correlation number (r) increases from 0.88 to 0.98. All R-values can be found in the Supplementary Table S3. **(B)** clustering dendrogram depicting the correlation, whereby the vertical axis represents the proteoform profiles of each donor and the horizontal scale represents the (dis)similarity between the samples.

Next, we aimed to evaluate the proteoform (dis)similarity of A1AT between donors and between human serum and milk of the same individual donor. To do this, the intensities of all peaks between 50,000 and 53,000 Da (Figure 2C) of all native MS spectra were extracted and subjected to a Pearson Correlation test. The resulting correlations between these proteoform profiles are plotted as a heatmap depicted in Figure 3A and the relativeness between samples is depicted in the dendrogram in Figure 3B. Notably, when comparing A1AT purified from a single donor’s serum and milk a very high correlation (*r* > 0.95) was observed, with the exceptions for donors 2 and 7. The low correlation observed for donor 2 is most likely due to the overlapping co-purified unknown protein within the same mass window in the milk sample. When removing the peak of 51,201.00 Da the Pearson Correlation coefficient increases from 0.88 to 0.98 (Supplementary Figure S3). The low correlation observed for donor 7 is most likely due to low A1AT concentration in the milk sample of this donor, a consequence of the fact that this sample was acquired at a late lactational state (Supplementary Table S1) (Chowanadisai and Lönnerdal, 2002; Zhu et al., 2021). Clearly, the intra-donor correlation between milk and serum A1AT is very high.

**FIGURE 4:**
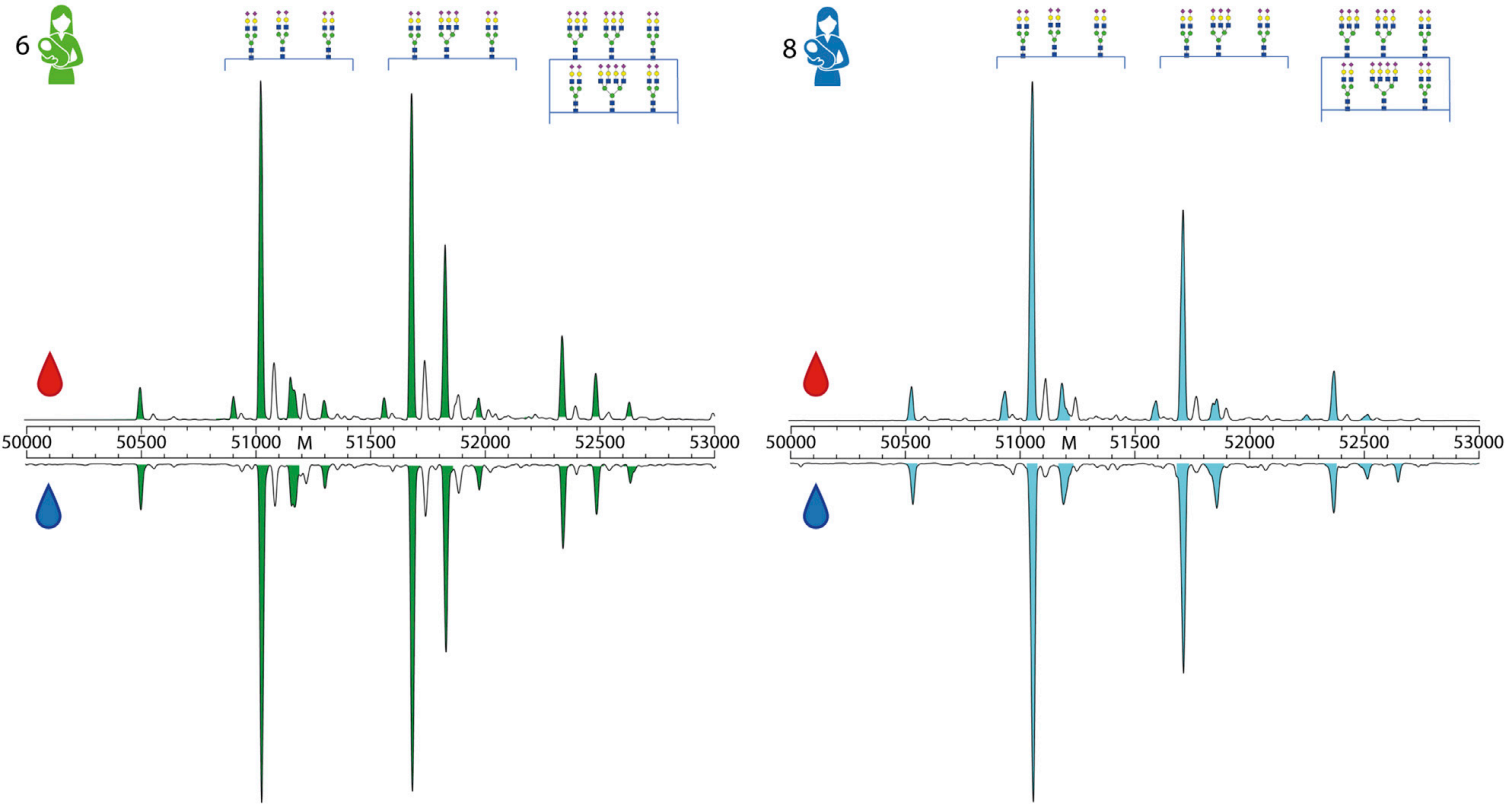
Direct comparison of proteoform profiles from donor 6 and 8. Depicted here are the zero-charge deconvoluted spectra of A1AT derived from serum (top, red droplet) and milk (bottom, blue droplet), in the mass range of 50,000–53,000 Da. Donors and peaks are color-coded as in Figure 1. The main peaks are annotated by their glycan composition, fully annotated proteoform profiles of these donors can be found in Supplementary Figures S7, S9. When comparing the abundances of different proteoforms between donor 6 and 8, it is clear that each profile is different. For example, donor 6 has a higher abundance of branched and fucocylated A1AT than donor 8. An overview of all measured proteoform profiles of all donors and all samples are given in the related Supplementary Figures S2–S9.

In contrast, the inter-donor correlation between A1AT proteoform profiles is much lower (Figures 3, 4). A main factor contributing to this low correlation is that many of these samples belong to A1AT of different homo- or heterozygote genotypes. Donors exhibiting the same genotype tend to have a higher inter-donor correlation, for example donors 4 and 5 and donors 7 and 8 had shared genotypes and were therefore more similar. Still, when examining the A1AT proteoform profiles it is evident that the distribution of different glycoforms is still substantially different between donors even if they exhibit the same genotype (Supplementary Figures S2–S9). Thus, we conclude that each of the donors has a rather unique A1AT proteoform profile, whereas within a donor the proteoform profiles between serum and milk A1AT are hardly distinguishable. The data revealing the donor specific proteoform profiles of A1AT for each donor is quite striking, albeit in full concordance with our earlier observations of donor specific proteoform profiles of serum fetuin/α-2-HS-Glycoprotein (Lin et al., 2019) and alpha-1 antichymotrypsin (AACT) (Čaval et al., 2021).

## Discussion

A1AT is a very versatile protein that is highly abundant in various biofluids and is considered as an acute phase protein. Its molecular function exceeds beyond its inhibitory proteolytic function, being also involved in many anti-inflammatory and immune-modulatory pathways. Although the function of A1AT has been extensively studied in serum, knowledge of the role of A1AT in human milk is limited to protecting other milk proteins from degradation by the gastrointestinal tract of the infant. However, it is well known to be a critical protein for infant health, as A1AT deficiency, a hereditary disorder causing extremely low A1AT concentrations, can cause chronic liver disease, cirrhosis, and liver failure in children and adults (Teckman et al., 2014; Greene et al., 2016). Most commonly, A1AT deficiency is the result of the Z (Glu366Lys) or S (Glu288Val) genotype and it is most severe in homozygous ZZ individuals. If the deficiency is left undiagnosed, which is possible as there is no standard screening practices at birth, then this can progress into liver and lung disease in adulthood (Teckman et al., 2014). Possibly, a better understanding of how to profile the genotypes of A1AT, such as the methods we used here, could even lead to better diagnosis in infancy. However, this does not change the fact that there are limited treatments for A1AT lung disease and no treatments for A1AT liver disease and patients often need transplantation.

A1AT is primarily synthesized in the liver hepatocytes, after which it is secreted into the serum. It has been suggested that A1AT in milk is synthesized locally, primarily based on the observation that complementary DNA of A1AT was detected in the mammary gland (Chowanadisai and Lönnerdal, 2002). Additionally, A1AT is suggested to exert different functions in human milk and serum. Therefore, we *a priori* hypothesized that the A1AT proteoform profiles would be distinctly different between milk and serum A1AT of an individual donor. This hypothesis was strengthened by the fact that such differences in proteoform profiles were already reported for the close-family protease inhibitor alpha-1-antichymotrypsin, when comparing AACT purified from human milk and serum (Urueña et al., 1998).

Using our personalized proteoform profiling approach for A1AT, we have here been able to closely compare the proteoform profiles of A1AT derived from human serum and milk within a single donor, evaluating in total eight donors. Conflicting with our original hypothesis, we found that proteoform profiles of A1AT from human milk and serum of a given donor were nearly identical. There were no observable differences in qualitative and quantitative *N*-glycosylation, and no differences in N-terminal processing and amount of cysteinylation. This can be theoretically explained in one of two ways. Option one, the synthesis of A1AT in different environments is highly controlled and conserved resulting in the extent of modifications and processing on A1AT being identical. Option two, A1AT in human milk primarily originates from the same source as A1AT in serum. This could occur *via* direct diffusion whereby the A1AT concentrations in human milk and serum are 0.1–0.4 mg/ml and 1.3 mg/ml respectively, suggesting a downward diffusion gradient (Chowanadisai and Lönnerdal, 2002). Notably, this is not the case for AACT, which has alike concentrations in human milk and serum of 0.4–7 and 0.4 mg/ml, respectively.

The first explanation is not likely, as this would mean that the cells producing the A1AT locally in the mammary gland should have a 100% identical glycosylation enzymatic machinery. As this machinery contains tens of enzymes all with their own abundance and activity in different cells, this is highly unlikely. Moreover, if the N-terminal processing would also occur locally, it is hard to explain why the efficiency of this process would also be perfectly identical when occurring either in the hepatocytes or in the A1AT producing cells in the mammary gland.

Thus, we conclude from our studies that most of the A1AT present in human milk from healthy donors originates from serum. A1AT could be diffused from serum and/or actively transported through a yet unknown mechanism. It is known that in the lungs A1AT is transported through transcytosis; more specifically bidirectional transcytosis across the endothelium and unidirectional transcytosis across the epithelium (Lockett et al., 2014). Future studies could focus on whether the same mechanisms also take place in the mammary gland, as the epithelial structure and organization of the two organs are quite similar. Gaining more information on these mechanisms should be valuable for mothers and/or infants who have been diagnosed with antitrypsin deficiency, and for mothers receiving antitrypsin therapy.

Finally, our research shows the potential to discover more about A1AT and its role in human milk. It is known that the A1AT concentration and proteoform profile in serum can change rapidly in response to an infection and/or inflammation. Our data suggest that altered A1AT profiles would also be reflected in human milk of lactating mothers (Lechowicz et al., 2020). It has also been shown that some of the immune-modulatory functions of A1AT depend on its glycosylation (McCarthy et al., 2014). As a result, changes in the proteoform profile of A1AT caused by inflammation in the mother could also have a direct anti-inflammatory effect on the infant. Additionally, our findings may have impact on augmentation/replacement therapy, which for instance is used as a treatment for patients with severe A1AT deficiency who have emphysema (Gøtzsche and Johansen, 2016). Treatment of these patients use A1AT proteins derived from the blood of healthy donors to increase the amount of A1AT in the lungs of patients with A1AT deficiency. Future studies and clinical practice should consider that patients who are breastfeeding with augmented A1AT profiles may also directly transfer these proteins to the infant *via* human milk.

In summary, by analyzing the in-depth proteoform profiles of alpha-1 antitrypsin extracted from either human serum or milk from several individual donors, we observed that each donor exhibits a unique A1AT proteoform profile. This proteoform profile is a signature of, and highly influenced by, the A1AT allotype of the donor, and directly reveals the homo- or heterozygote nature of the donor. However, for donors of exactly the same allotype the A1AT proteoform profiles are still rather distinctive, due to donor-specific differences in *N*-glycosylation, N-terminal processing and cysteinylation.

Strikingly, considering these differences, we found that the proteoform profiles of A1AT extracted from either human serum or milk from the same donor were nearly indistinguishable. This suggests that the processes leading to A1AT *N*-glycosylation, N-terminal processing/truncation and cysteinylation are “identical” for the A1AT present in serum and milk. From this we can only conclude that practically all A1AT present in human serum and milk originates from the same source, namely the liver, and thus that A1AT present in milk gets there *via* direct transmission from the serum. This conclusion is in contrast to previous reports, but may also have consequences for A1AT replacement therapy. Moreover, it should then also be considered that changes in the A1AT serum profile of the mother, for instance as a consequence of an inflammatory event, will likely lead to changes in the A1AT milk profile. Downstream consequences to infant health from altered A1AT milk profiles are not known.

## Data Availability

The datasets presented in this study can be found in online repositories. The names of the repository/repositories and accession number(s) can be found below: https://massive.ucsd.edu/ProteoSAFe/static/massive.jsp, MSV000088705.
