# Supplementary material for: Proteoform Profiles Reveal That Alpha-1-Antitrypsin in Human Serum and Milk Is Derived From a Common Source
Source: Front Mol Biosci. 2022 Feb 22;9:858856. doi: 10.3389/fmolb.2022.858856 (PMC8902301; doi:10.3389/fmolb.2022.858856)
Supplement: Supplementary file 1 [file DataSheet1.PDF]

# Supplementary information, Figures and Tables

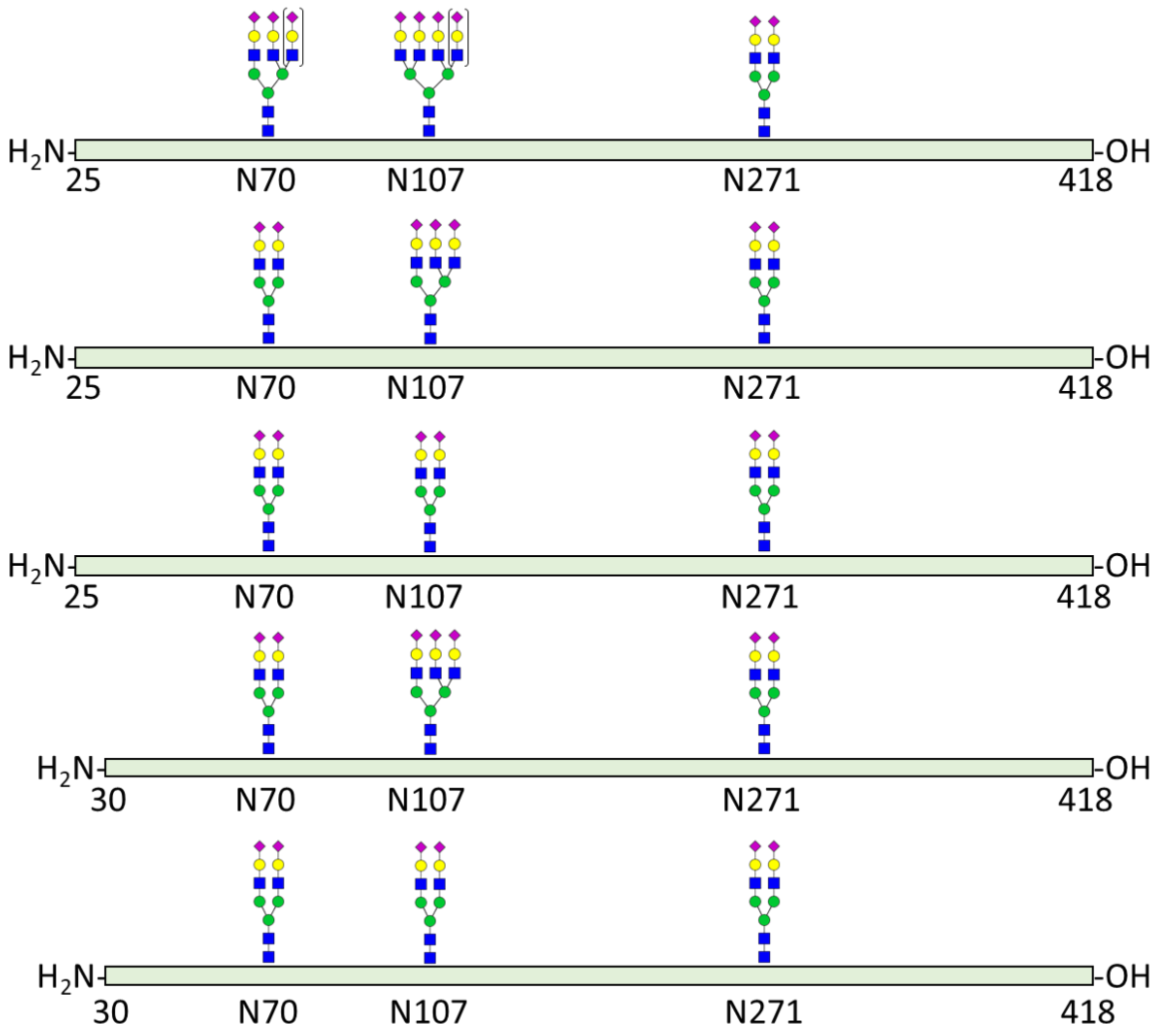


**Supplemental Figure 1: Schematic overview of the observed most abundant proteoforms of A1AT.** The main difference between the different proteoforms are caused by the extend of branching on the *N*-glycosites N107 and N70 and the 5 amino acid truncation at the N-terminus, which is in agreement with previous reports (4,24–26). The glycans additionally can be fucosylated on the core GlcNAc and on the branch GlcNAc.

**Supplemental Table S1:** Average masses used to annotate the observed post translational modifications (PTMs)

| PTM | Mass shift (Da) |
|---|---|
| N-terminal truncation (-EDPQG) | 526.50 |
| Hexose/Manose/Galactose | 162.1424 |
| *N*-acetylhexosamine/*N*-acetylglycosamine | 203.1950 |
| Deoxyhexose/fucose | 146.1430 |
| Decysteinylation | -119.1421 |

**Supplemental Table S2:** Information on the donors.

| Donor number | Maternal age in years | Gestation in weeks | Gestation in days | Sample collection weeks postpartum | Days between milk and serum collection | Genotype |
|---|---|---|---|---|---|---|
| 1 | 36 | 40 | 4 | 7 | 2 | M1VM2 |
| 2 | 33 | 41 | 1 | 6 | 1 | M1AM1V |
| 3 | 37 | 40 | 3 | 6 | 0 | M3 |
| 4 | 34 | 39 | 0 | 6 | 0 | M1A |
| 5 | 38 | 41 | 4 | 8 | 1 | M2 |
| 6 | 34 | 41 | 0 | 7 | 0 | M1V |
| 7 | 30 | 41 | 0 | 40 | 0 | M1A |
| 8 | 33 | 40 | 5 | 17 | 0 | M1V |

**Supplemental Table 3:** All r numbers of the Pearson Correlation. The number of the sample corresponds to the donor number indicated in Supplemental Table 2.

| | serum_8 | serum_7 | serum_6 | serum_5 | serum_4 | serum_3 | serum_2 | serum_1 | milk_8 | milk_7 | milk_6 | milk_5 | milk_4 | milk_3 | milk_2 | milk_1 |
|---|---|---|---|---|---|---|---|---|---|---|---|---|---|---|---|---|
| Milk_1 | 0.46 | 0.51 | 0.85 | 0.84 | 0.85 | 0.01 | 0.57 | 0.99 | 0.46 | 0.58 | 0.85 | 0.85 | 0.86 | -0.01 | 0.52 | 1.00 |
| Milk_2 | 0.64 | 0.63 | 0.58 | 0.60 | 0.61 | 0.02 | 0.88 | 0.63 | 0.65 | 0.62 | 0.59 | 0.60 | 0.59 | 0.05 | 1.00 | |
| Milk_3 | 0.00 | -0.01 | -0.01 | -0.01 | -0.01 | 0.97 | -0.01 | -0.01 | 0.01 | 0.03 | -0.01 | 0.00 | -0.01 | 1.00 | | |
| Milk_4 | -0.01 | 0.03 | 0.99 | 0.98 | 0.96 | 0.02 | 0.66 | 0.79 | -0.01 | 0.19 | 0.98 | 0.95 | 1.00 | | | |
| Milk_5 | 0.00 | 0.01 | 0.94 | 0.95 | 0.97 | 0.02 | 0.64 | 0.76 | -0.01 | 0.19 | 0.94 | 1.00 | | | | |
| Milk_6 | -0.01 | 0.03 | 0.98 | 0.96 | 0.97 | 0.03 | 0.65 | 0.83 | -0.01 | 0.19 | 1.00 | | | | | |
| Milk_7 | 0.85 | 0.84 | 0.17 | 0.20 | 0.18 | 0.03 | 0.60 | 0.59 | 0.84 | 1.00 | | | | | | |
| Milk_8 | 0.95 | 0.95 | -0.01 | -0.01 | -0.01 | 0.59 | 0.67 | 0.46 | 1.00 | | | | | | | |
| Serum_1 | 0.48 | 0.52 | 0.83 | 0.85 | 0.85 | 0.00 | 0.57 | 1.00 | | | | | | | | |
| Serum_2 | 0.71 | 0.70 | 0.64 | 0.68 | 0.68 | 0.00 | 1.00 | | | | | | | | | |
| Serum_3 | -0.01 | -0.01 | 0.02 | 0.00 | 0.00 | 1.00 | | | | | | | | | | |
| Serum_4 | -0.01 | 0.03 | 0.96 | 0.99 | 1.00 | | | | | | | | | | | |
| Serum_5 | -0.01 | 0.03 | 0.98 | 1.00 | | | | | | | | | | | | |
| Serum_6 | -0.01 | 0.03 | 1.00 | | | | | | | | | | | | | |
| Serum_7 | 0.98 | 1.00 | | | | | | | | | | | | | | |
| Serum_8 | 1.00 | | | | | | | | | | | | | | | |

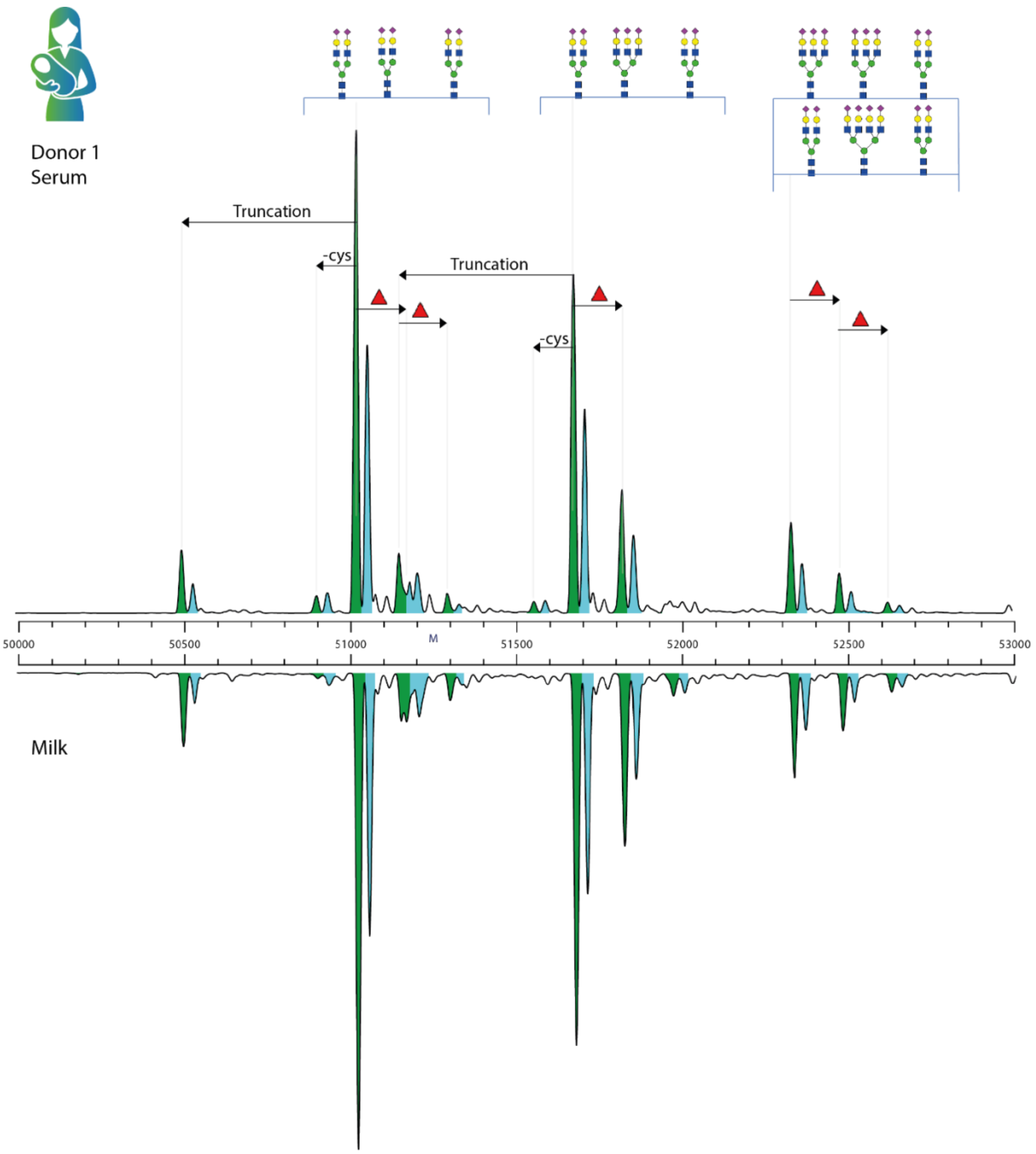


**Supplemental Figure 2: Annotated proteoform profiles of A1AT derived from human serum and milk from donor 1.** Depicted is the zero-charge deconvoluted native mass spectrum of A1AT derived from serum (top) and milk (bottom), in the mass range from 50 to 53.5 kDa. Colored peaks represent peaks originating from the same genotype, green and blue for the genotypes M2 and M1V, respectively. The main peaks are annotated by their glycan composition and indicated with arrows are the additional annotated PTMs: the N-terminal truncation (truncation), decysteinylation of C256 (-cys), and additional fucoses (red triangle) which can either be located on the antennae or on the glycan core.

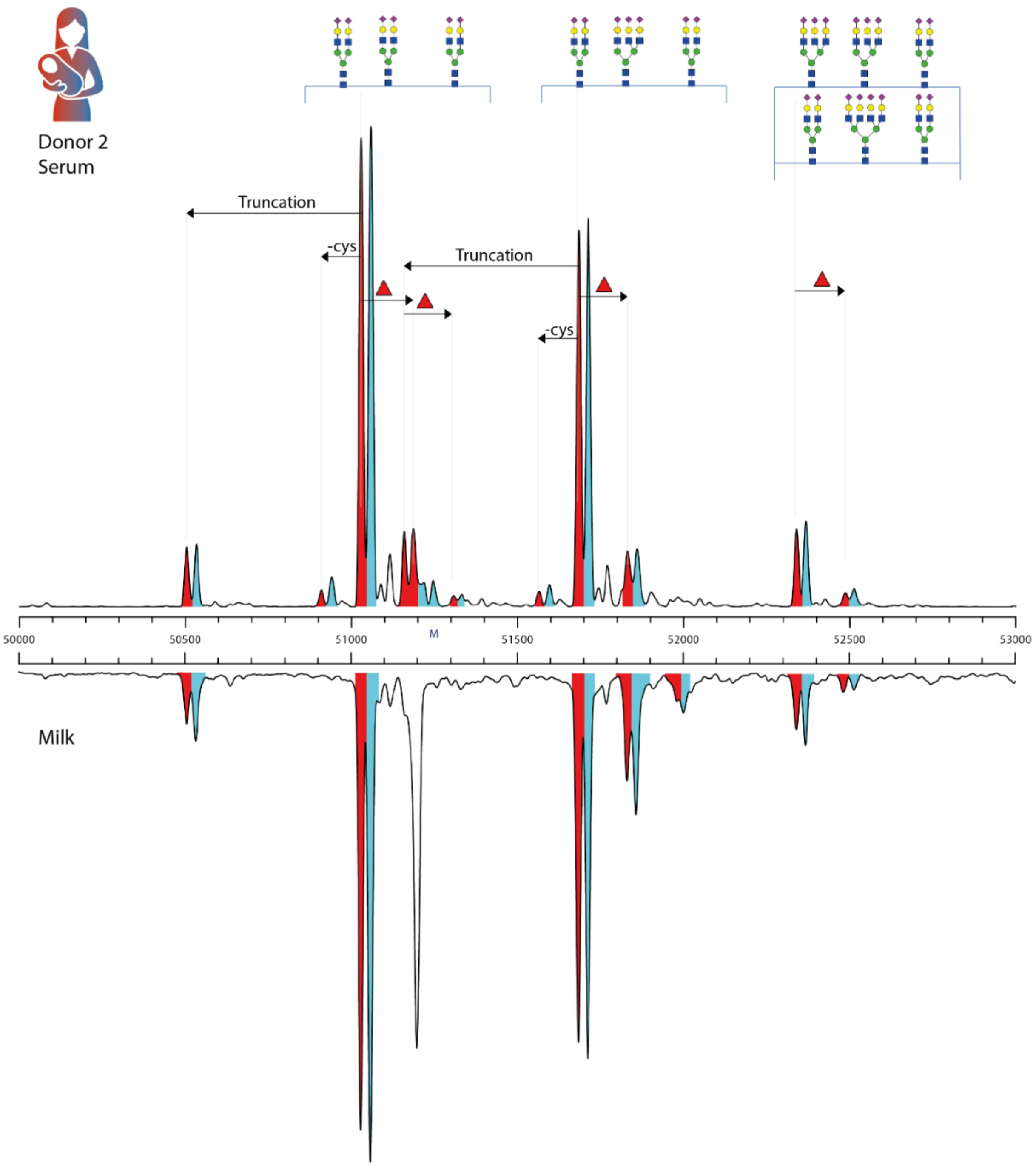


**Supplemental Figure 3: Annotated proteoform profiles of A1AT derived from human serum and milk from donor 2.** Depicted is the zero-charge deconvoluted native mass spectrum of A1AT derived from serum (top) and milk (bottom), in the mass range from 50 to 53.5 kDa. Colored peaks represent peaks originating from the same genotype, red and blue for M1A and M1V, respectively. The main peaks are annotated by their glycan composition and indicated with arrows are the additional annotated PTMs: the N-terminal truncation (truncation), decysteinylation of C256 (-cys), and additional fucoses (red triangle) which can either be located on the antennae or on the glycan core. The Pearson correlation coefficient is 0.88, however, when disregarding the peak of the ~51200 Da contaminant, this increases to 0.98.

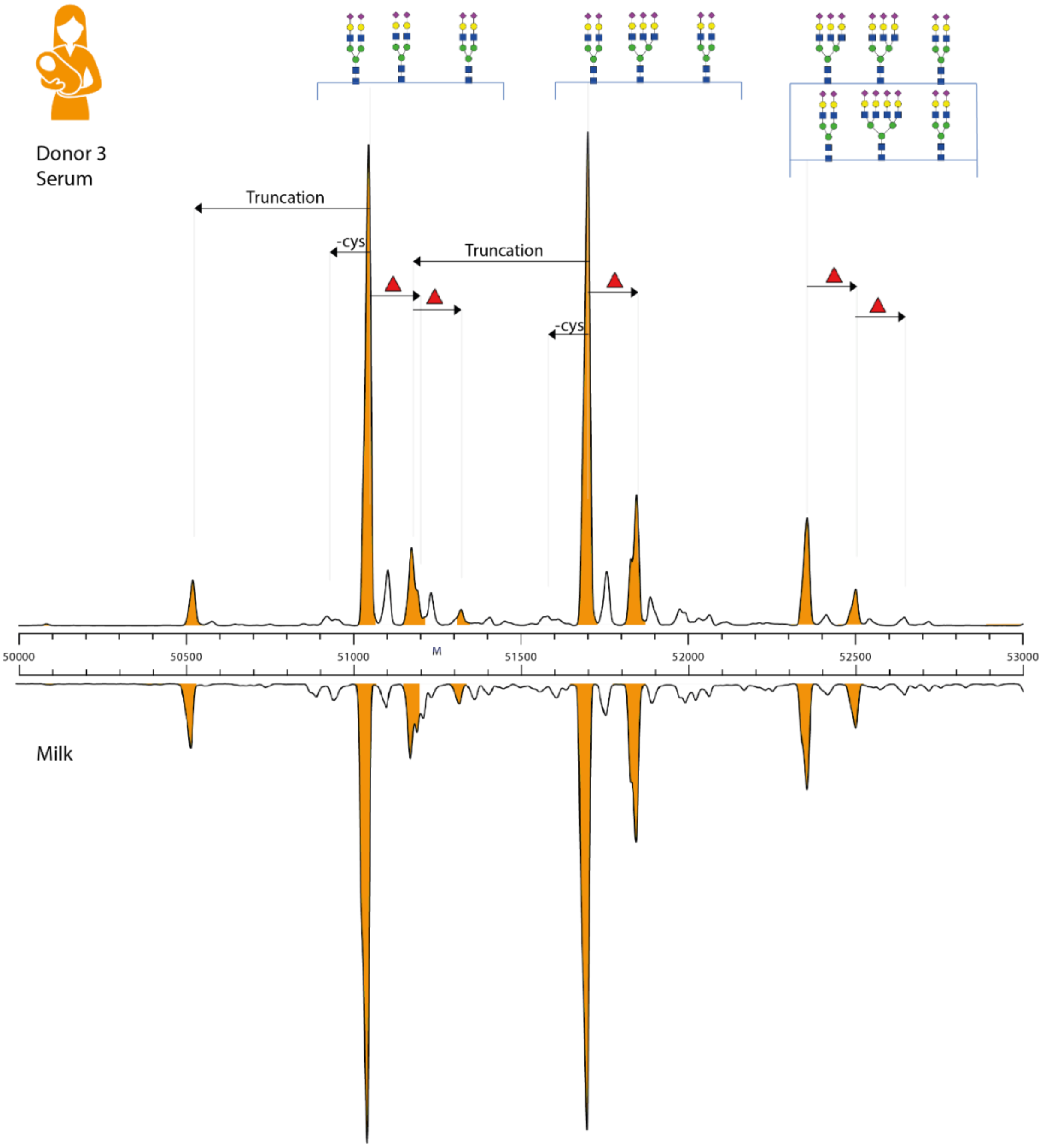


**Supplemental Figure 4: Annotated proteoform profiles of A1AT derived from human serum and milk from donor 3.** Depicted is the zero-charge deconvoluted native mass spectrum of A1AT derived from serum (top) and milk (bottom), in the mass range from 50 to 53.5 kDa. Colored peaks represent peaks originating from the same genotype, orange for M3. The main peaks are annotated by their glycan composition and indicated with arrows are the additional annotated PTMs: the N-terminal truncation (truncation), and additional fucoses (red triangle) which can either be located on the antennae or on the glycan core.

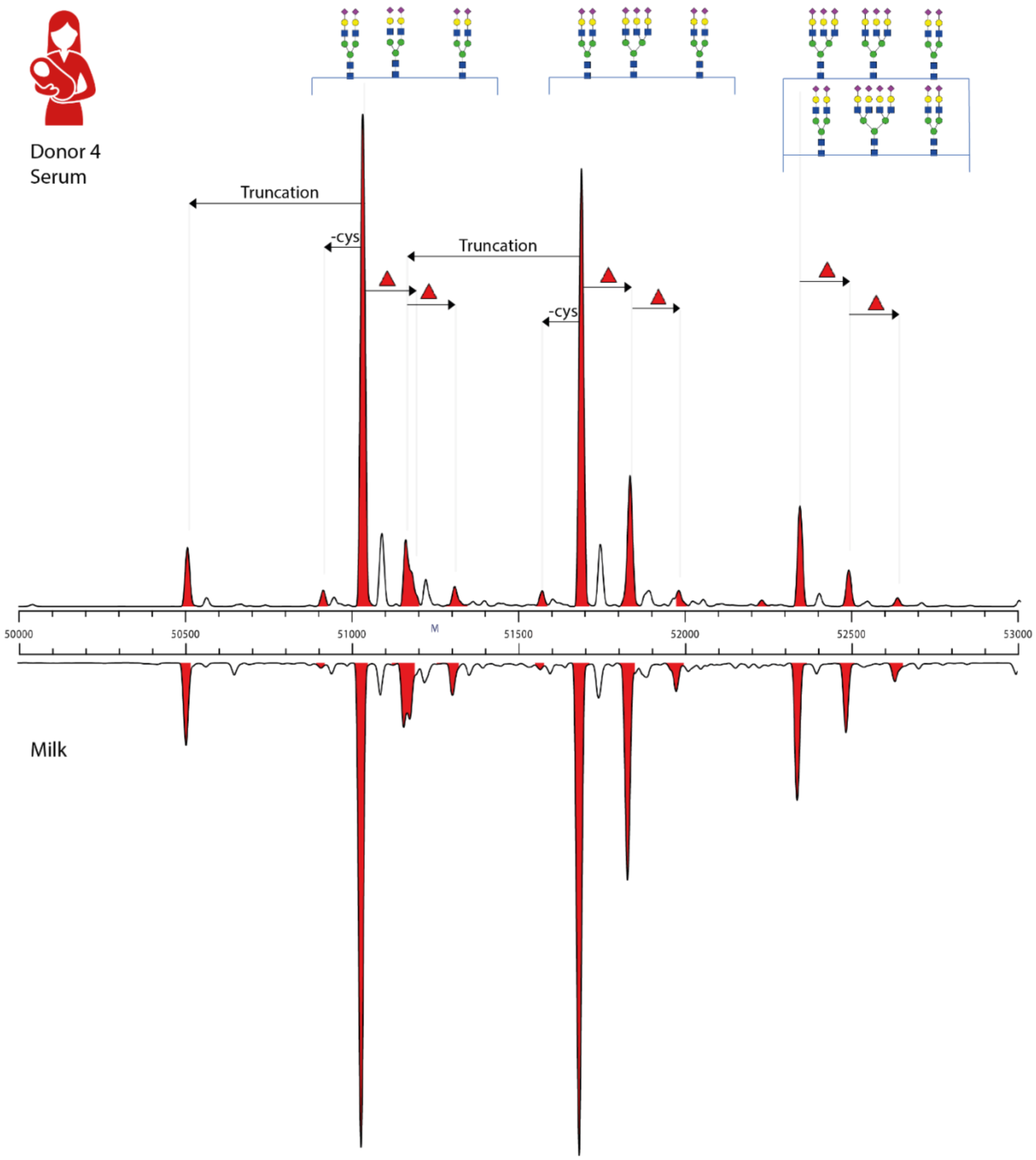


**Supplemental Figure 5: Annotated proteoform profiles of A1AT derived from human serum and milk from donor 4.** Depicted is the zero-charge deconvoluted native mass spectrum of A1AT derived from serum (top) and milk (bottom), in the mass range from 50 to 53.5 kDa. Colored peaks represent peaks originating from the same genotype, red for M1A. The main peaks are annotated by their glycan composition and indicated with arrows are the additional annotated PTMs: the N-terminal truncation (truncation), and additional fucoses (red triangle) which can either be located on the antennae or on the glycan core.

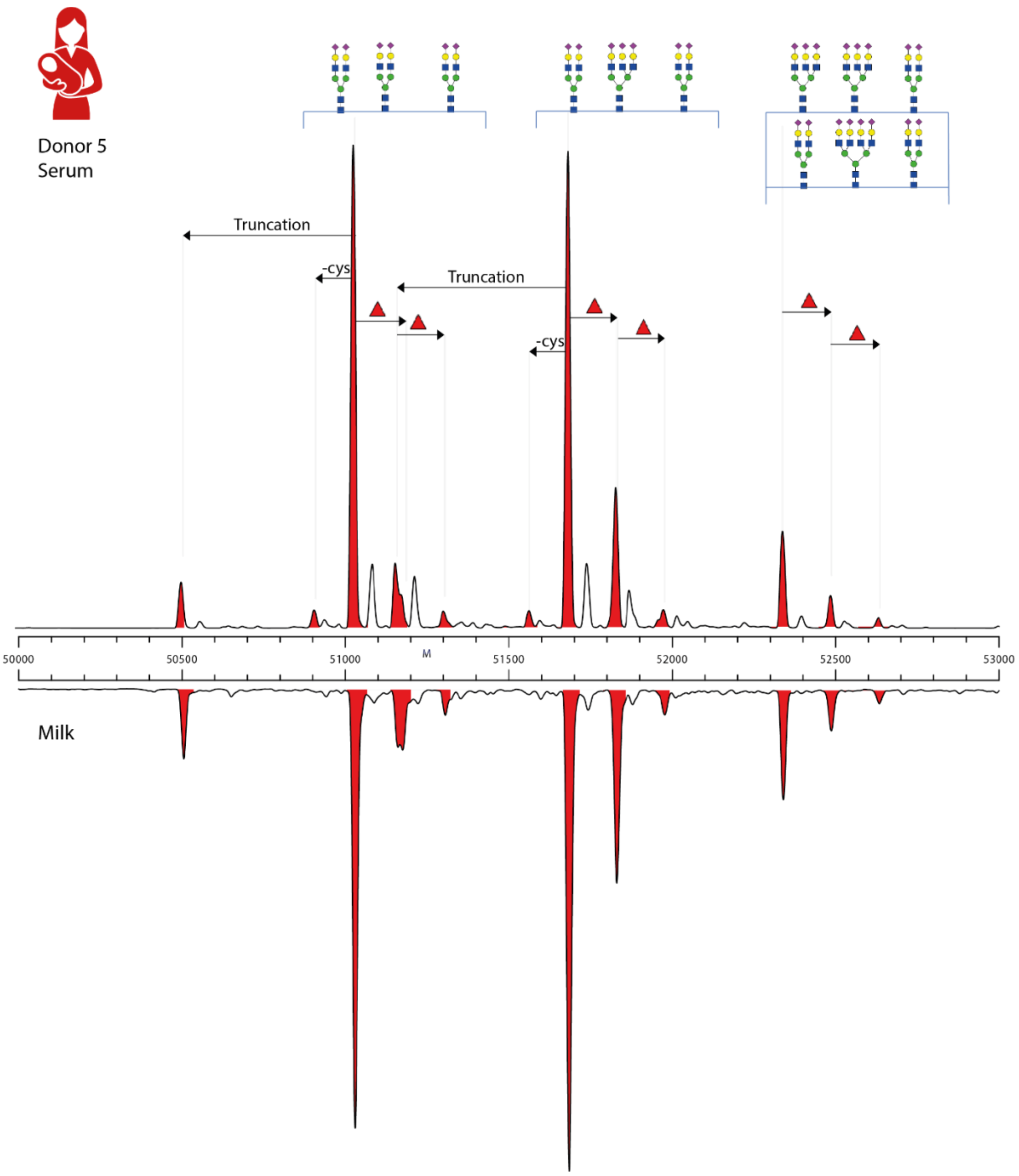


**Supplemental Figure 6: Annotated proteoform profiles of A1AT derived from human serum and milk from donor 5.** Depicted is the zero-charge deconvoluted native mass spectrum of A1AT derived from serum (top) and milk (bottom), in the mass range from 50 to 53.5 kDa. Colored peaks represent peaks originating from the same genotype, red for M1A. The main peaks are annotated by their glycan composition and indicated with arrows are the additional annotated PTMs: the N-terminal truncation (truncation), and additional fucoses (red triangle) which can either be located on the antennae or on the glycan core.

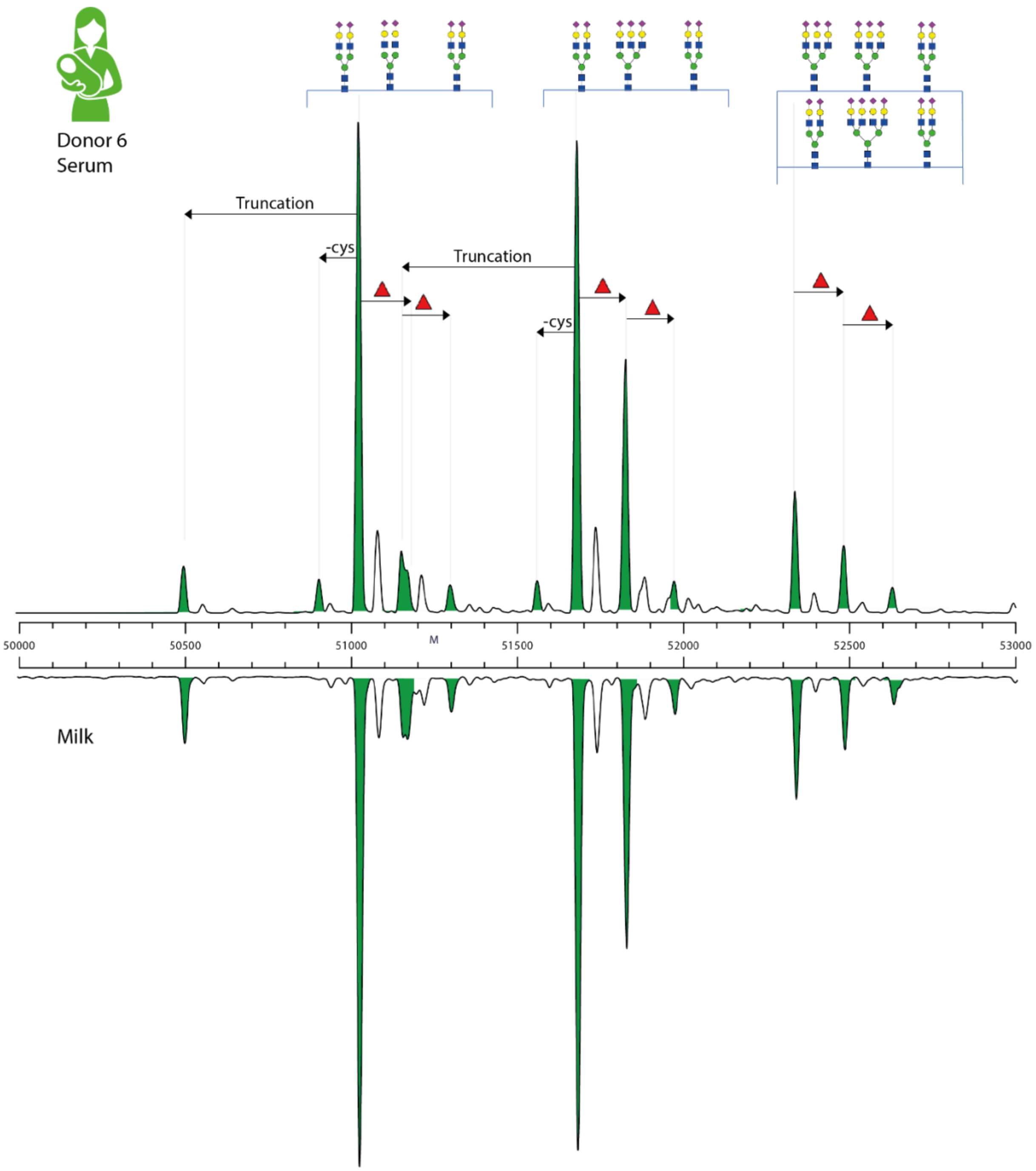


**Supplemental Figure 7: Annotated proteoform profiles of A1AT derived from human serum and milk from donor 6.** Depicted is the zero-charge deconvoluted native mass spectrum of A1AT derived from serum (top) and milk (bottom), in the mass range from 50 to 53.5 kDa. Colored peaks represent peaks originating from the same genotype, green for M2. The main peaks are annotated by their glycan composition and indicated with arrows are the additional annotated PTMs: the N-terminal truncation (truncation), and additional fucoses (red triangle) which can either be located on the antennae or on the glycan core.

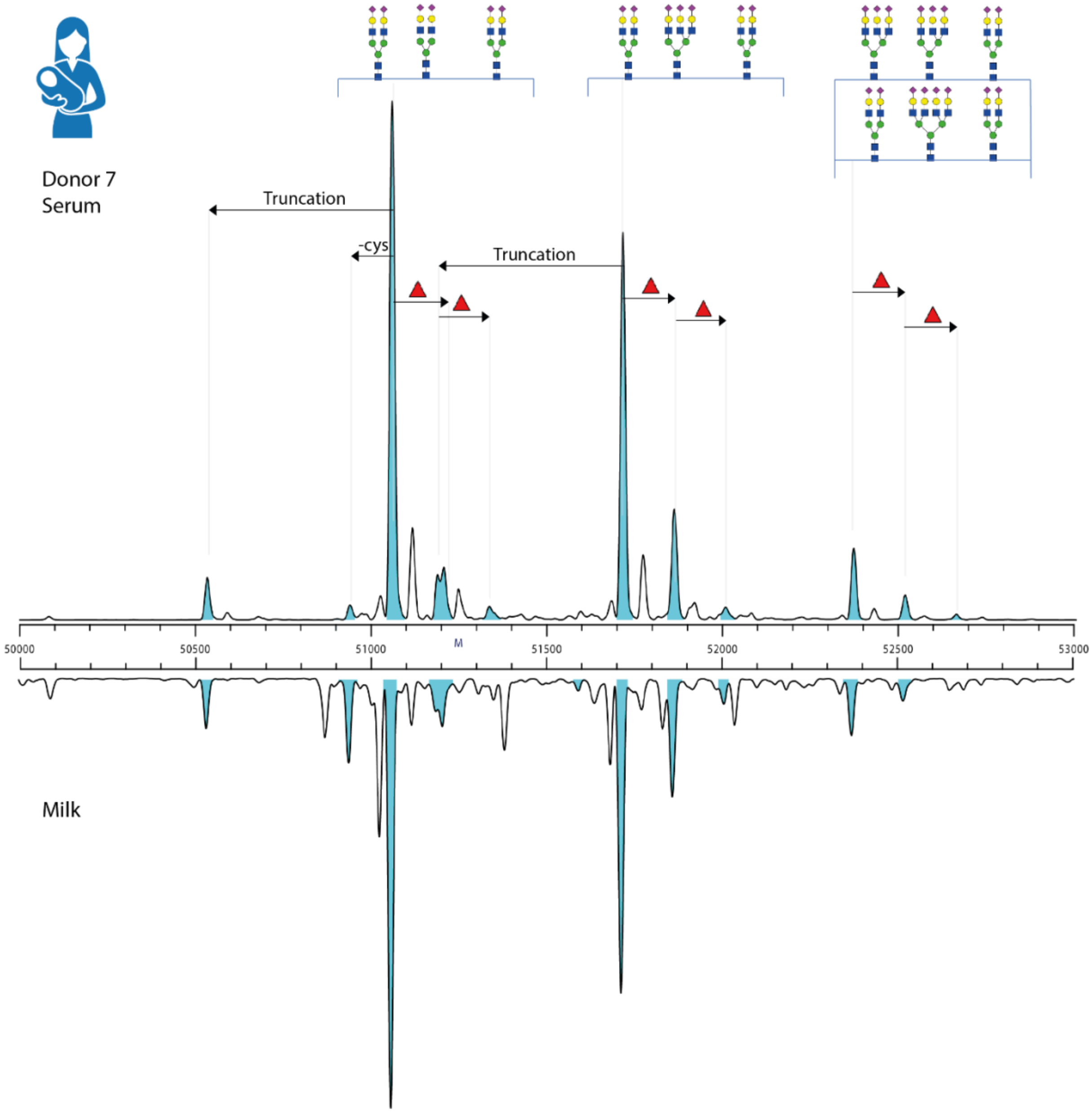


**Supplemental Figure 8: Annotated proteoform profiles of A1AT derived from human serum and milk from donor 7.** Depicted is the zero-charge deconvoluted native mass spectrum of A1AT derived from serum (top) and milk (bottom), in the mass range from 50 to 53.5 kDa. Colored peaks represent peaks originating from the same genotype, blue for M1V. The main peaks are annotated by their glycan composition and indicated with arrows are the additional annotated PTMs: the N-terminal truncation (truncation), and additional fucoses (red triangle) which can either be located on the antennae or on the glycan core.

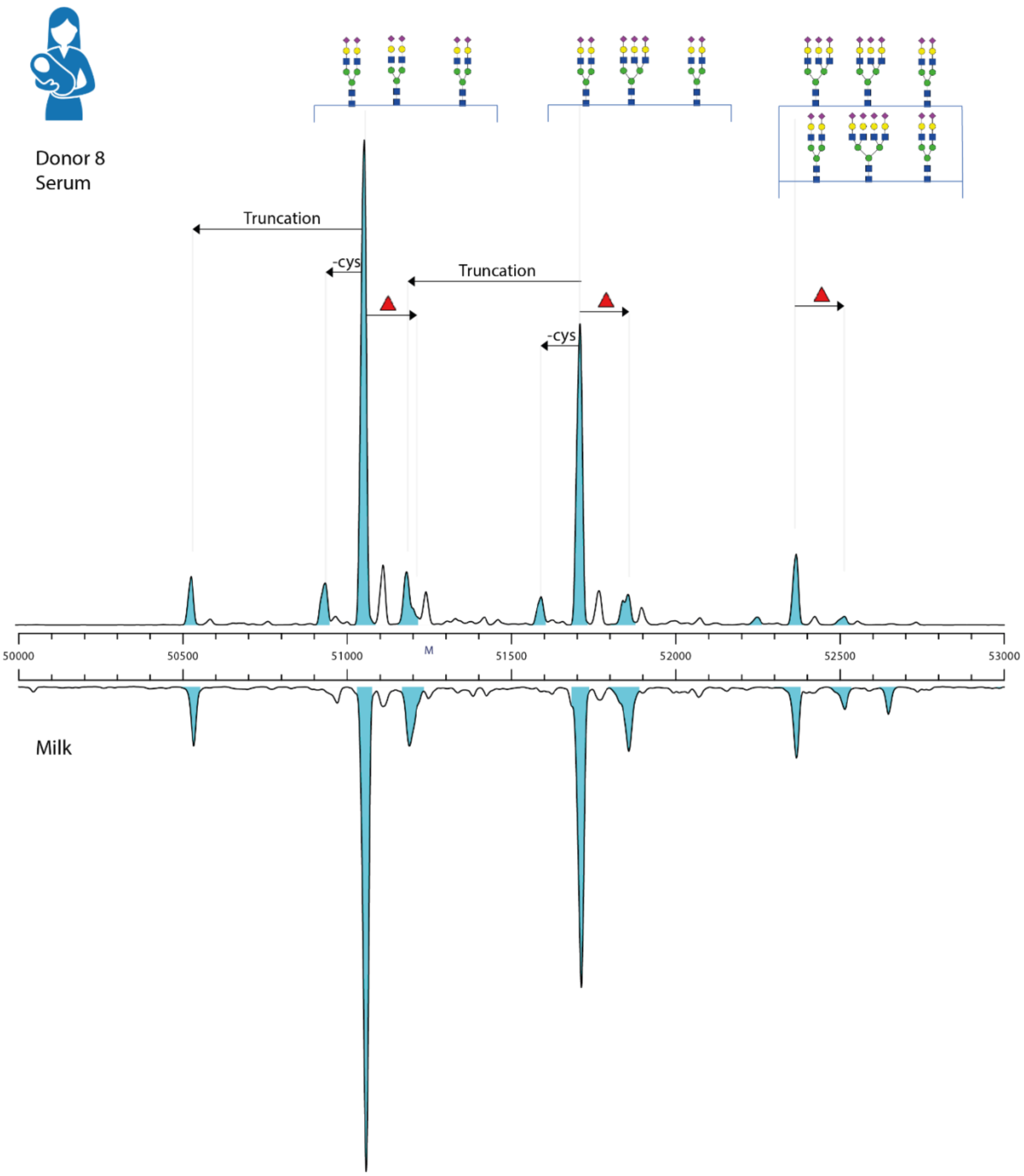


**Supplemental Figure 9: Annotated proteoform profiles of A1AT derived from human serum and milk from donor 8.** Depicted is the zero-charge deconvoluted native mass spectrum of A1AT derived from serum (top) and milk (bottom), in the mass range from 50 to 53.5 kDa. Colored peaks represent peaks originating from the same genotype, blue for M1V. The main peaks are annotated by their glycan composition and indicated with arrows are the additional annotated PTMs: the N-terminal truncation (truncation), and additional fucoses (red triangle) which can either be located on the antennae or on the glycan core.
